# Exploring Trust in Research Among Black American Men at a Health Promotion Symposium in Rural North Carolina

**DOI:** 10.1007/s10900-024-01399-6

**Published:** 2024-09-06

**Authors:** Shawnta L. Lloyd, Kelvin Lamonte Williams, Goldie S. Byrd

**Affiliations:** Maya Angelou Center for Health Equity, Wake Forest University School of Medicine, 525 Vine Street, Suite # 150, Winston Salem, NC 27101 USA

**Keywords:** Trust in research, Faith-based engagement, Rural health, Black or African American, Men’s health, Health research participation

## Abstract

Despite efforts to diversify research and health programs, Black American men remain a “hard-to-reach” population while collectively suffering from some of the worst health outcomes in the United States. Faith- and community-based approaches have shown potential to engage Black Americans in health promotion and health research activities. The purpose of this article is to examine health research participation and trust in research among a sample of Black American men in rural North Carolina who attended a community-based health symposium, culturally tailored for Black American men (*n* = 112). A cross-sectional survey was administered among men to learn about health status, health concerns, and perception of health research. Among 106 men who completed the survey, most reported no prior participation in health research (68.87%), but almost a third of men reported interest in participating in health research. No significant differences in trust in research was found based on interest in research participation (interested in research participation, not interested in research participation, no response), presenting an opportunity to increase the trustworthiness of medical institutions and build relationships with this population. These findings will inform future research and health programming for Black American men in rural locations.

**Supplementary Information:**

The online version contains supplementary material available at 10.1007/s10900-024-01399-6.

## Introduction

In the United States, Black Americans are underrepresented in clinical research and suffer from some on the poorest health outcomes. Despite the development of the National Institutes of Health (NIH) Revitalization Act in 1993 [1] and increases in the racial and ethnic diversity of clinical research [2], the racial and ethnic diversity of clinical research has fallen short. Black Americans represent nearly 14% of the population in the United States [3], and only represent about 6% of NIH-funded clinical research [2]. The lack of participation of Black Americans in health research may alter the generalizability of research findings, treatments, and cures, impeding opportunities for improvement in health outcomes. Although Black Americans have experienced decreases in mortality rates in recent decades, this population is still at a higher risk for chronic diseases such as hypertension and diabetes compared to their White counterparts [4]. The increased morbidity among Black Americans has been linked to social determinants of health (e.g. poverty, incarceration, racial discrimination, violence, environmental exposures, and lack of healthcare access) and risk factors for chronic disease and adverse health outcomes such as poor nutrition, physical inactivity, hypertension, tobacco smoking and substance abuse [5].

Compared to Black American women, Black American men suffer from even worse health outcomes within the Black American community, burdened by homicide, unintentional injuries, cancer, and heart disease, the number one cause of death among Black American men of all ages [6]. At birth, Black American men are expected to live an average of 66.7 years, on average, which is more than 10 years shorter than the national average life expectancy and more than 8 years shorter than Black American women [7]. Further, Black American men who reside in rural areas have the lowest life expectancy in the United States [8] and are at an increased risk of heart failure [9]. Poor health outcomes in rural areas have been attributed to issues such as increased risk factors for death and disease (e.g. tobacco use, obesity, low seatbelt use, and hypertension), less access to healthcare, poverty, and insufficient healthcare supplies and health professionals [10, 11]. Identifying innovative methods to engage with and build trust with Black American men to increase participation in health programming and research is vital to improve health outcomes.

Previous efforts have been successful in engaging men in health programs and research through faith-based organizations [12–15]. Historically, the church has been a respected and trusted agency in the Black American community. It is not simply a venue where congregants worship and receive spiritual guidance. The Black American church has long been involved in political action, community outreach, and the social and economic welfare of the community [16]. Faith-based approaches have shown notable potential to engage Black American communities in health promotion and health research activities [12, 13, 17–20]. For instance, the Maya Angelou Center for Health Equity (MACHE) built a network of more than 100 faith leaders across North Carolina called the Triad Pastors Network (TPN) [21]. This network includes an advisory board of faith leaders that engage at least quarterly with the Center to provide direction for community-informed research projects and community events. The collaboration between MACHE and the TPN has produced products such as community webinars that have reached over 300,000 people, a research project on the perspectives of Black Americans on COVID-19 [22, 23], engagement projects to increase awareness about men’s health [24], and a congregational health ambassadors program that assists churches in enhancing or establishing their own health ministries.

Black American men in rural areas face a unique set of barriers that put them at risk for adverse health. The church remains a staple in the Black American community to engage with the local community, especially in rural areas where places to gather may be limited. Despite efforts to diversify research and health programs, Black American men remain a “hard-to-reach” population while collectively suffering from some of the worst health outcomes in the United States. The purpose of this article is to examine health research participation and trust in research among a sample of Black American men in rural North Carolina who attended a community-based health promotion event.

## Materials and Methods

### A Men’s Prayer Breakfast and Health Symposium

During Men’s Health month, MACHE at the Wake Forest University School of Medicine collaborated with the United Shiloh Missionary Baptist Association (USMBA) and the TPN to host a men’s prayer breakfast and health symposium in a Tier 1 county in rural North Carolina [25]. In the state of North Carolina, counties are ranked as Tier 1, 2, or 3, with Tier 1 counties being the most distressed, based on unemployment rate, median household income, population growth, and adjusted property tax base per capita. The purpose of this event was to create a health space for Black American men to (1) raise awareness of health issues affecting Black American men and health research participation, and (2) link men to health resources. Using a community-based participatory research approach [26], teammates from MACHE, TPN, and USMBA formed an advisory board of 10 volunteer members to plan and implement the event. The board met bi-weekly for 3 months. Major health topics to be presented at the event were selected by community members on the board, and community members on the board were included in each aspect of the event planning.

On the day of the event, attendees were served breakfast by a local caterer before engaging in the health program. During the health program, attendees were welcomed by and engaged with local community leaders. The highlights of the event included a keynote address by a Black American male psychoneuroendocrinologist, who provided a dynamic presentation on general men’s health. The keynote address was followed by a health panel, consisting of Black American physicians. A local retired judge moderated a heath panel of 3 local male physicians with specialties in family medicine, cardiology, and urology. This panel discussed the topics of diabetes, prostate cancer, and heart health. Following the initial presentations from the panel, attendees were able to engage with members of the panel and ask their own health questions of concern. The program closed with a presentation from a local pastor who addressed the relationship between health and spirituality. Throughout the event, local health vendors were on site and disseminated health information to attendees.

### Recruitment Strategies

The population of interest to attend this event included Black or African American men, 18 years of age or older. Nonetheless, any male 18 years of age or older was welcomed to attend the event. Faith-based methods were largely used to inform Black American men in the community about the event and register men to attend the event. Recruitment strategies included word of mouth, flyers, and media announcements.

#### Word of Mouth

Church Ambassadors: The USMBA includes a membership of 42 faith leaders in the geographic location of interest. To garner the support of the community for the event and ease the registration process for men in the community, faith leaders who were members of the USMBA were asked to select a church ambassador. The duties of this church ambassador included informing men in their church about the event and the benefits of participating as well as helping the men register for the event, if interested in attending. Ambassadors received a visa gift card for recruiting men to attend the event and encouraging invited attendees to complete the event survey.

Boots on the Ground Efforts: Members of the Board put forth tireless efforts to inform the community of the event via word of mouth. Meetings and phone calls were conducted with local faith leaders and community leaders to inform them of the upcoming event and to ask these individuals to spread the word to their networks. Some members of the board were invited to provide announcements during church or community events to spread the word about the event.

#### Flyers

The Board worked together to develop culturally relevant flyers for Black American men in the geographic area of interest. It was not only important to visually represent Black American men on the flyer, but it was also important to include leaders that men in the community were familiar with and trusted. Black American male faith leaders and physicians were included in flyers disseminated in the community. Flyers were distributed through church services, church listservs, social media pages, and boots on the ground efforts.

#### Media Announcements

Press releases for local newspapers, scripts for radio announcements, and a video announcement were also disseminated to inform the community about the event. The press release was published in 4 local newspapers. The announcement for the event was disseminated through 2 local radio stations. In addition to the re-occurring radio announcements, a local pastor and faith leader completed 2 interviews with local radio stations to spread the word about the event. A 60-second video announcement was also developed with the event flyer and disseminated to local churches to share during worship services and special events.

### Attendees

A total of 112 men attended the event, and 14 Black American male speakers were present (not included in analysis). Approximately 38% of the men in attendance were walk-ups (i.e., registered on-site the day of the event). Men were represented from 11 counties across North Carolina and one county in South Carolina. The mean age of attendees was about 63 years of age. More than 90% of men in attendance reported being non-Hispanic and the remaining proportion of men did not report a response. Nearly 96% of men in attendance reported being Black or African American. The remaining men reported being American Indian or Alaskan Native, Other, or provided no information on race. Most men learned of the event from a church or faith leader (60%), followed by a flyer or poster (12%), other source (7%), social media page (4%), and email (4%). Approximately 13% of men in attendance did not report how they were informed of the event.

### Measures

After attending the program, male attendees were asked to complete a cross-sectional survey. Questions were derived from studies in the extant literature [27–31]. Data were collected on demographic information, self-reported health information, trust in health research, willingness to participate in health research, and feedback about the event. Demographic data included age (numerical data), gender (female, male, other), Hispanic, Latino, or Spanish descent (yes, no), race (American Indian or Alaska Native, Asian, White, Black or African American, Native Hawaiian or Other Pacific Islander, Other), highest level of education (less than high school, high school diploma/GED, associate degree or trade school, bachelor degree, master degree, doctoral/professional degree), marital status (married, widowed, divorced, separated, single (never married), and employment status (work at a full-time/part-time job, disabled, retired, unemployed, in school/training, other). Attendees reported information about their health including top health concerns (open-ended), self-reported health status (excellent, very good, good, fair, poor), health insurance status (yes, no), past 12-month physical exam (yes, no), past 12-month dental exam (yes, no), having a trusted healthcare provider (yes, no), organ donor status (yes, no), and regular care or assistance to a friend or family member with a disability or health problem in the past 30 days (caregiver; yes, no). The survey also included questions about past research participation (Have you ever participated in a health research study or a clinical trial? ) and interest in participating in research (Are you interested in participating in a health research study or a clinical trial? ). Responses to research questions were dichotomous (yes, no). Attendees were asked “Would you volunteer to participate in a health research study if you were asked to …” participate in specific research activities, including complete a survey about your health, complete a memory and thinking test, have your medical records reviewed, give a blood sample, provide spinal fluid by having a needle inserted into your spinal canal (spinal tap), take medication, use medical equipment, complete a brain scan, stay overnight in a hospital or clinic, be in a genetic study, and donate your brain to research after death. The trust section (9 items) of the Perceptions of Research Trustworthiness (PoRT) scale was included in the survey to assess trust in research [27]. Last, attendees were asked to provide feedback on the event activities.

### Data Analysis

All data was managed in REDCap^®^ (Research Electronic Data Capture) and analyzed in SAS ^®^ 9.4. Descriptive statistics were calculated for demographic, health, and research participation. The mean and standard deviation were calculated for age. The responses for the 9 items in the PoRT Trust scale were graded on a Likert scale (1- strongly disagree, 2- disagree, 3- neither agree or disagree, 4- agree, 5- strongly agree). The mean score and standard deviation for each item and for overall trust in research was calculated [27]. Due to missing responses for some of the items in the PoRT scale, the mean was imputed for missing values in each statement by interest in research. For example, among respondents who were interested in participating in research, missing values for statement 1 of the PoRT scale were replaced with the mean values derived from respondents who answered the statement. For the remaining categorical variables, the frequency of a response and the percentage were calculated. The scores from the PoRT scale and the proportions from the types of research activities respondents were willing to participate in were stratified by the respondents’ interest in participating in research (interested, not interested, and no response). For the PoRT scale, an analysis of variance (ANOVA) was conducted to determine any significant differences between the 3 groups. For the types of research activities, a Fisher’s exact test was conducted to assess the association between interest in health research and types of research activities due to the small cells counts (< 5) for some variables. Attendees were asked open-ended questions about their top health concerns. Reviewers read through all responses to become familiar with the data. Responses were then categorized.

## Results

### Demographic Summary

A total of 106 men completed the survey at the event. Table 1 shows a demographic summary of survey respondents. The mean age of respondents was 62.43 years of age; respondents ranged from 20 to 91 years of age. Approximately 3% of respondents reported being of Hispanic, Latino, or Spanish descent, and nearly 85% of respondents reported being Black or African American. More than 80% of respondents had received at least a high school diploma or GED, and more than half of respondents reported being married. About 29% of respondents reported working either part-time or full-time and almost 39% reported being retired.

**Table 1:** Sociodemographic Summary of Event Attendees

Characteristics	Frequency	Percent
Age (M, SD) *	62.43	15.44
Gender		
Male	94	88.68
Other	1	0.94
No Response	11	10.38
Hispanic, Latino, or Spanish descent		
No	89	83.96
Yes	3	2.83
No Response	14	13.21
Race		
American Indian or Alaska Native	4	3.77
Black or African American	90	84.91
Other	1	0.94
No Response	11	10.38
Highest Level of Education		
Less than High School	7	6.60
High School Diploma/GED	37	34.91
Associate Degree/Trade School	19	17.92
Bachelor Degree	11	10.38
Master Degree	14	13.21
Doctoral/Professional Degree	6	5.66
No Response	12	11.32
Marital Status		
Married	64	60.38
Widowed	2	1.89
Divorced	14	13.21
Separated	3	2.83
Single (Never Married)	12	11.32
No Response	11	10.38
Employment		
Work at a Full-time/Part-time Job	31	29.25
Disabled	13	12.26
Retired	41	38.68
Unemployed	1	0.94
In School/Training	3	2.83
Other	5	4.72
No Response	12	11.32

*n = 93, M = Mean, SD = Standard Deviation

### Self-Reported Health

The greatest proportion of respondents self-reported their health as good (45.28%), followed by very good (29.25%) and fair (18.87%; Table 2). Most respondents reported having health insurance (92.45%), receiving a physical exam in the past 12 months (85.85%) and receiving a dental exam in the past 12 months (69.81%). Only about 11% of respondents reported that they did not have a healthcare provider they trusted with their medical care. In addition to caring for their own needs, 37.74% of respondents reported that they had provided regular care or assistance to a family member or friend within the past 30 days. The majority of respondents also reported that they were not an organ donor (67.92%). The top 3 answers listed under top health concerns among respondents included blood pressure, diabetes, and general health.

**Table 2:** Self-reported Health Information of Event Attendees

Health Information	Frequency	Percent
Self-reported Health Status		
Excellent	5	4.72
Very Good	31	29.25
Good	48	45.28
Fair	20	18.87
Poor	0	0.00
No Response	2	1.89
Health Insurance		
No	5	4.72
Yes	98	92.45
No Response	3	2.83
Past 12-month Physical Exam		
No	14	13.21
Yes	91	85.85
No Response	1	0.94
Past 12-month Dental Exam		
No	30	28.30
Yes	74	69.81
No Response	2	1.89
Trusted Health Care Provider		
No	12	11.32
Yes	87	82.08
No Response	7	6.60
Caregiver		
No	43	40.57
Yes	40	37.74
No Response	23	21.70
Organ Donor		
No	72	67.92
Yes	29	27.36
No Response	5	4.72

### Participation of Health Research

**Fig. 1:**
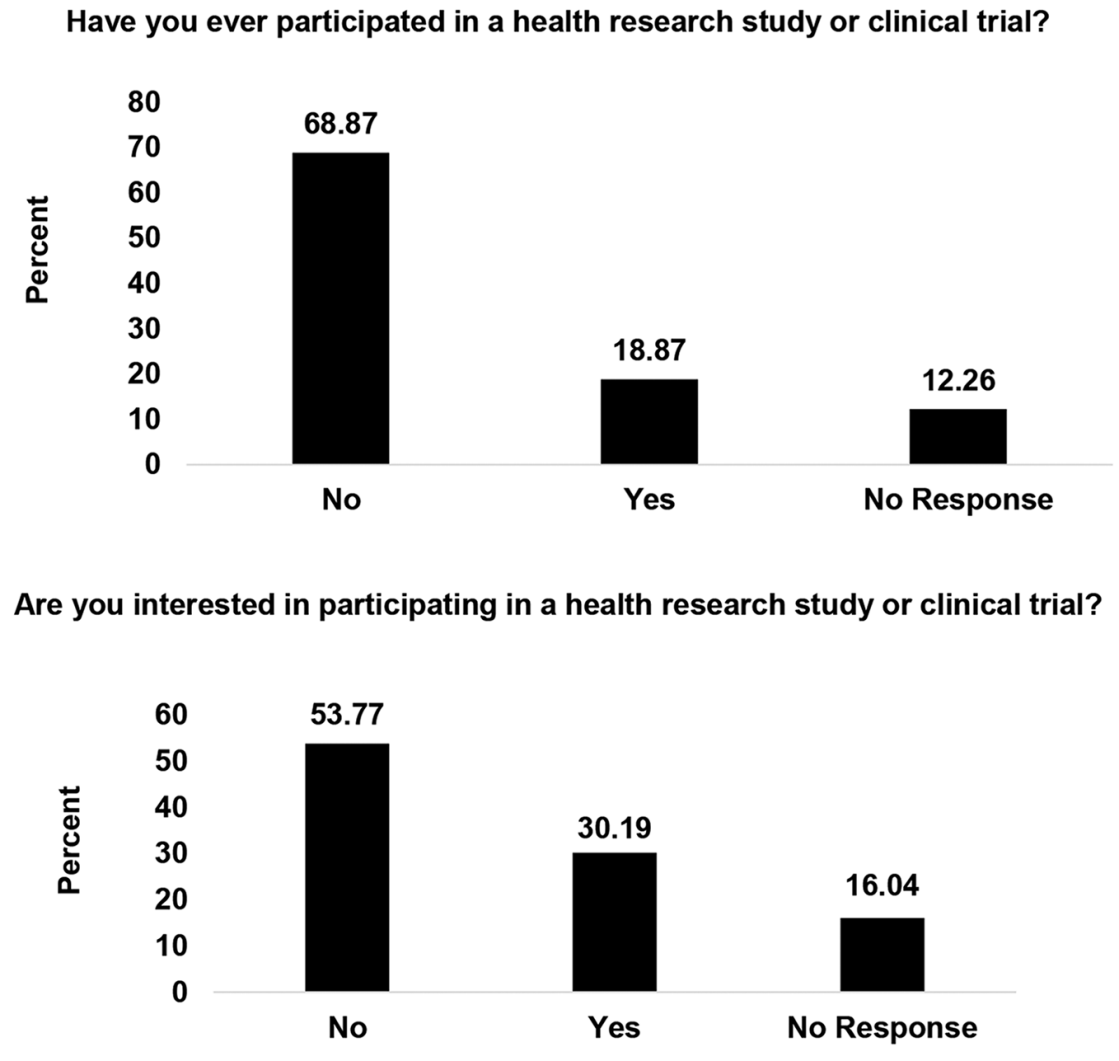
Research Participation among Event Attendees, n = 106. *Note* These percentages presented in Fig. 1 are representative of a total of 106 respondents. Frequency of responses can be found in supplemental documents

When asked about previous participation in a health research study or clinical trial, 18.87% of respondents reported participation in health research in the past (*n* = 20; Fig. 1). Most respondents reported that they had never participated in health research (*n* = 73; 68.87%). Further, respondents were asked if they would be interested in participating in a health research study or clinical trial. Almost a third of respondents reported that they would be interested in participating in health research (*n* = 32; 30.19%).

**Fig. 2:**
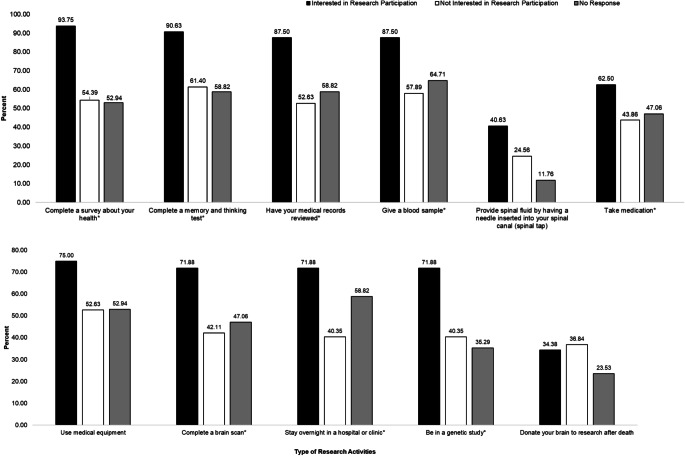
Willingness to Participate in Research Activities by Interest in Health Research Participation, n = 106. *p < 0.05; *Note* These percentages presented in Fig. 2 are representative of a total of 106 respondents. Frequency of responses can be found in supplemental documents

Respondents were asked what types of research activities they would be willing to participate in. When stratified by interest in research, there were no significant differences in respondents’ willingness to complete a spinal tap, use medical equipment, or donate their brain to research after death (Fig. 2). Compared to respondents who were not interested in research and those who did not respond, a significantly greater proportion of respondents who were interested in research participation were willing to participate in research activities such as taking a health survey, completing memory or thinking tests, having a medical record review, providing a blood sample, taking medication, completing a brain scan, staying overnight in a hospital, and participating in a genetic study.

Trust in research did not differ among respondents based on their interest in participating in health research based on results from the PoRT scale (Table 3). However, compared to respondents who were interested in research or did not respond to the interest in research question, respondents who had no interest in participating in research reported significantly lower trust scores when asked to report how much they agreed with the following statements: “Participation in medical research benefits society;” “If I had a chance to be in a medical research study, it would be easy for me to decide to join in or not;” and “If I had a chance to be in a medical research study, I would be sure that participating in medical research would be the best choice for me.”

**Table 3:** Trust in Research among Event Attendees

PoRT Statements	Interested in Research *n* = 32 M (SD)	Not Interested in Research *n* = 57 M (SD)	No Response *n* = 17 M (SD)
Overall Trust Score	3.63 (0.58)	3.51 (0.63)	3.49 (0.60)
Medical researchers tell people everything they need to know about being in a research study.	2.90 (1.15)	3.22 (1.13)	2.85 (0.93)
Any info about me that I give to medical researchers would be kept confidential.	3.67 (0.96)	3.70 (1.06)	3.23 (1.42)
Medical researchers would never give someone something that would hurt them, just to study how it works in people.	3.10 (1.20)	3.30 (1.08)	2.67 (0.96)
Participation in medical research benefits society.*	4.10 (0.89)	3.65 (0.92)	4.17 (0.60)
Medical researchers usually tell people in a research study about different things they could do to get well.	3.97 (0.54)	3.73 (0.76)	4.00 (0.61)
If I had a chance to be in a medical research study, it would be easy for me to decide to join in or not.*	4.10 (0.86)	3.38 (0.85)	3.67 (0.89)
Medical researchers only do research on people who know it is happening.	3.00 (1.19)	3.06 (1.02)	3.75 (1.13)
My physician would not ask me to be in a medical research study if they thought it would hurt me.	3.62 (1.00)	3.84 (0.66)	4.00 (0.50)
If I had a chance to be in a medical research study, I would be sure that participating in medical research would be the best choice for me.*	4.19 (0.69)	3.72 (0.76)	3.49 (0.60)

*p < 0.05

## Discussion

In this article, we described health research participation and trust in research among a sample of Black American men in rural North Carolina. More than 100 men attended a community-based event, Raising the Bar for Black Men’s Health and Wellness. Most respondents had not participated in health research or clinical trials, but about 30% of respondents were interested in participating in health research. Regardless of their interest in health research, there were no significant differences in trust in research among respondents.

Although the majority of men who attended the event reported being involved in the healthcare system, few men had ever participated in health research, and merely a third of the men were interested in participating in research. These findings are beneficial in guiding future research and developing health programming tailored to Black American men in rural areas. These results point to the question of why these men have never participated in research and what factors are driving men to have an interest or disinterest in health research participation. Although barriers and facilitators of research participation among Black Americans have been thoroughly examined in the extant literature [32–35], few studies focus on the unique barriers and facilitators of Black American men and strategies to engage Black American men in research. Previous studies that focused on prostate cancer have found that barriers to research participation among Black American men included factors such as medical and research mistrust, transportation, invasive procedures, time commitments, and lack of knowledge about the disease being studied [13, 36]. A facilitator for Black American men’s participation in health research was being at risk for the disease of interest, monetary compensation, and civic duty [13, 36, 37].

There is a need to increase the trustworthiness of medical and research institutions to engage and build trusting, authentic relationships with this “hard-to-reach” population. In general, the trust measured among this sample of Black American men was relatively neutral. There was not a strong trust in research, but there was also not a strong mistrust in research. To develop and sustain trust among Black American men during engagement for health and research programs, strategies such as culturally-tailored marketing, community involvement, and partnering with local organizations have been suggested [13]. Raising the Bar for Black Men’s Health and Wellness partnered with local faith organizations to reach Black American men in a rural area and recruited Black American men to attend the event through culturally tailored methods and materials. Continued community involvement and engagement with Black American men in rural North Carolina through community events and the dissemination of information may foster trust between an academic center and Black American men in the community.

These analyses have limitations that should be noted. The responses collected about health research participation and trust in research via the cross-sectional survey represent the thoughts and opinions of a convenience sample of Black American men and may not be generalizable to all Black American men who reside in rural geographic areas. A strong faith-based approach was utilized to recruit Black American men to participate in this health event. Some Black American men who are not a part of a church may not have been reached. Nonetheless, the team reached out to Black American men via newspapers, social media, radio, and in the general community. Although efforts were made to reach Black American men who may not be a part of a church, some Black American men may not consider themselves spiritual or religious, and these individuals may feel uncomfortable attending an event marketed as a “prayer breakfast and health symposium” or being in a faith environment. Anecdotally, our team learned that other events, including several funerals took place during the same date as the men’s health event; therefore, some men were not able to attend the event due to scheduling conflicts. In addition, even though attendees at the event were as young as 20 years of age, the majority of attendees were middle-aged and older adults. This sample also consisted of men who largely considered themselves in good health and had access to health insurance and trusted healthcare professionals. To thoroughly engage Black American men in rural areas to understand facilitators to research participation, increase health research participation, and build trust in health research, there is a need to reach diverse groups of men within the Black American population including men across the lifespan, those who may be non-spiritual or non-religious men, and men who may lack access to medical resources.

Despite limitations, an academic and faith-based partnership was established to successfully bring more than 100 Black American men together in a rural area, enabling our team to learn about health research participation and trust in research among this specific group of men. Through these efforts, our team engaged with populations that are underrepresented in health research and health programming, Black American men in rural geographic locations. Further, using an enhanced understanding of Black American men’s’ health and research concerns, future engagement and research activities with Black American men in this geographic location and other rural areas in North Carolina can be tailored to meet the community needs. Additionally, our team can use Black Americans men’s perception of research to begin to develop future projects that aid in learning how we can increase trust in health research as well as personnel who work within these institutions.

## Electronic Supplementary Material

Below is the link to the electronic supplementary material.

Supplementary Material 1

## References

[1] Institute of Medicine (US) Committee on Ethical and Legal Issues Relating to the Inclusion of Women in Clinical Studies, Mastroianni, A. C., Faden, R., & Federman, D. (Eds.). (1994). *Women and health research: Ethical and legal issues of including women in clinical studies: Volume I* (pp. 103–43). National Academies Press (US). NIH Revitalization Act of 1993, Public Law. https://www.ncbi.nlm.nih.gov/books/NBK236531/25144026

[2] Ma, M. A., Gutiérrez, D. E., Frausto, J. M., & Al-Delaimy, W. K. (2021). Minority representation in clinical trials in the United States: Trends over the past 25 years. *Mayo Clinic Proceedings*, *96*(1), 264–266. 10.1016/j.mayocp.2020.10.027 33413830 10.1016/j.mayocp.2020.10.027

[3] U.S. Census Bureau QuickFacts: United States. (n.d.). Retrieved August 21 (2023). from https://www.census.gov/quickfacts/fact/table/US/IPE120221

[4] National Center for Chronic Disease Prevention and Health Promotion. (2017, July 3). African American health. *Centers for Disease Control and Prevention*. Retrieved August 21, 2023, from https://www.cdc.gov/vitalsigns/aahealth/index.html

[5] Noonan, A. S., Velasco-Mondragon, H. E., & Wagner, F. A. (2016). Improving the health of African americans in the USA: An overdue opportunity for social justice. *Public Health Reviews*, *37*(1), 12. 10.1186/s40985-016-0025-4 29450054 10.1186/s40985-016-0025-4 PMC5810013

[6] Center for Disease Control and Prevention (2022, March 2). From the CDC-Leading Causes of Death-Males all races and origins 2018. *Centers for Disease Control and Prevention*. Retrieved October 25, 2022, from https://www.cdc.gov/minorityhealth/lcod/men/2018/nonhispanic-black/index.htm

[7] Arias, E., Tejada-Vera, B., Kochanek, K. D., & Farida, B. A. (2022). *Provisional life expectancy estimates for 2021* (No. no 23). Hyattsville, MD: National Center for Health Statistics. Retrieved from 10.15620/cdc:118999

[8] Improving health among Black men in rural South Carolina - College of Nursing | University of South Carolina. (n.d.). Retrieved August 21 (2023). from https://www.sc.edu/study/colleges_schools/nursing/news/2023/menshealth.php

[9] Turecamo, S. E., Xu, M., Dixon, D., Powell-Wiley, T. M., Mumma, M. T., Joo, J., … Roger, V. L. (2023). Association of rurality with risk of heart failure. *JAMA Cardiology*, *8*(3), 231. 10.1001/jamacardio.2022.5211. 10.1001/jamacardio.2022.5211 PMC9878434 36696094

[10] Caldwell, J. T., Ford, C. L., Wallace, S. P., Wang, M. C., & Takahashi, L. M. (2016). Intersection of living in a rural Versus Urban Area and Race/Ethnicity in explaining Access to Health Care in the United States. *American Journal of Public Health*, *106*(8), 1463–1469. 10.2105/AJPH.2016.303212 27310341 10.2105/AJPH.2016.303212 PMC4940644

[11] Office of Rural Health. (2023, May 11). About rural health. *Center for Disease Control and Prevention*. Retrieved August 21, 2023, from https://www.cdc.gov/ruralhealth/about.html

[12] Saunders, D. R., Holt, C. L., Le, D., Slade, J. L., Muwwakkil, B., Savoy, A., … Naslund, M. J. (2015). Recruitment and participation of African American men in church-based health promotion workshops. *Journal of Community Health*, *40*(6), 1300–1310. 10.1007/s10900-015-0054-9. 10.1007/s10900-015-0054-9 26089253

[13] Rogers, C. R., Matthews, P., Brooks, E., Le Duc, N., Washington, C., McKoy, A., … Fetters, M. D. (2021). Barriers to and facilitators of recruitment of adult African American men for colorectal cancer research: An instrumental exploratory case study.*JCO Oncology Practice*, *17*(5), e686–e694. 10.1200/OP.21.00008. 10.1200/OP.21.00008 PMC8258132 33974818

[14] Woods, V. D., Montgomery, S. B., & Herring, R. P. (2004). Recruiting Black/African American men for research on prostate cancer prevention. *Cancer*, *100*(5), 1017–1025. 10.1002/cncr.20029 14983498 10.1002/cncr.20029

[15] Graham, L. F., Scott, L., Lopeyok, E., Douglas, H., Gubrium, A., & Buchanan, D. (2018). Outreach strategies to recruit low-income African American men to participate in health promotion programs and research: Lessons from the men of color health awareness (MOCHA) project. *American Journal of Men’s Health*, *12*(5), 1307–1316. 10.1177/1557988318768602 29695204 10.1177/1557988318768602 PMC6142128

[16] Lincoln, C. E., & Mamiya, L. H. (1990). *The Black Church in the African American experience*. Duke University Press. 10.2307/j.ctv125jv2p

[17] McNeill, L. H., Reitzel, L. R., Escoto, K. H., Roberson, C. L., Nguyen, N., Vidrine, J. I., … Wetter, D. W. (2018). Engaging black churches to address cancer health disparities: Project CHURCH. *Frontiers in Public Health*, *6*. Retrieved from https://www.frontiersin.org/articles/10.3389/fpubh.2018.00191. 10.3389/fpubh.2018.00191 PMC6060541 30073158

[18] Campbell, M. K., Hudson, M. A., Resnicow, K., Blakeney, N., Paxton, A., & Baskin, M. (2007). Church-based health promotion interventions: Evidence and lessons learned. *Annual Review of Public Health*, *28*, 213–234. 10.1146/annurev.publhealth.28.021406.144016 17155879 10.1146/annurev.publhealth.28.021406.144016

[19] Majee, W., Anakwe, A., Onyeaka, K., Laboy, V., Mutamba, J., Shikles, M., & Chen, L. W. (2023). Participant perspectives on the effects of an African American faith-based health promotion educational intervention: A qualitative study. *Journal of Racial and Ethnic Health Disparities*, *10*(3), 1115–1126. 10.1007/s40615-022-01299-2 35394621 10.1007/s40615-022-01299-2 PMC8992409

[20] Hippolyte, J. M., Phillips-Caesar, E. G., Winston, G. J., Charlson, M. E., & Peterson, J. C. (2013). Recruitment and retention techniques for developing faith-based research partnerships, New York City, 2009–2012. *Preventing Chronic Disease*, *10*(E30). 10.5888/pcd10.120142 10.5888/pcd10.120142 PMC3603629 23469766

[21] Gwathmey, T. M., Williams, K. L., Caban-Holt, A., Starks, T. D., Foy, C. G., Mathews, A., & Byrd, G. S. (2024). Building a community partnership for the development of Health ministries within the African American Community: The Triad Pastors Network. *Journal of Community Health*. 10.1007/s10900-023-01315-4 38265538 10.1007/s10900-023-01315-4 PMC10981582

[22] Foy, C. G., Lloyd, S. L., Williams, K. L., Gwathmey, T. M., Caban-Holt, A., Starks, T. D., … Byrd, G. S. (2023). Gender, age and COVID-19 vaccination status in African American adult faith-based congregants in the Southeastern United States. *Journal of Racial and Ethnic Health Disparities*. 10.1007/s40615-023-01744-w. 10.1007/s40615-023-01744-w 37580437

[23] Lloyd, S. L., Foy, C. G., Caban-Holt, A., Gwathmey, T., Williams, K. L., Starks, T. D., … Byrd, G. S. (2023). Assessing the role of trust in public health agencies and COVID-19 vaccination status among a community sample of African Americans in North Carolina. *Journal of Racial and Ethnic Health Disparities*. 10.1007/s40615-023-01646-x. 10.1007/s40615-023-01646-x PMC10241131 37273163

[24] Lloyd, S. L., Williams, K. L., Network, P., Caban-Holt, T., Craft, A., Baker, S. D., L., & Byrd, G. S. (2024). The Black men’s health forum: Improving health knowledge and willingness to participate in research. *Health Education & Behavior*, *51*(1), 104–112. 10.1177/10901981231206074 37905517 10.1177/10901981231206074

[25] North Carolina Department of Commerce. (n.d.). County Distress Rankings (Tiers). Retrieved May 1 (2023). from https://www.commerce.nc.gov/grants-incentives/county-distress-rankings-tiers

[26] Israel, B. A., Schulz, A. J., Parker, E. A., Becker, A. B., & Community-Campus Partnerships for Health. (2001). Community-based participatory research: Policy recommendations for promoting a partnership approach in health research. *Education for Health (Abingdon England)*, *14*(2), 182–197. 10.1080/13576280110051055 14742017 10.1080/13576280110051055

[27] Stallings, S. C., Cunningham-Erves, J., Frazier, C., Ichimura, J. S., Hurd, T. C., Jurinsky, J., … Wilkins, C. H. (2022). Development and validation of the perceptions of research trustworthiness scale to measure trust among minoritized racial and ethnic groups in biomedical research in the US. *JAMA network open*, *5*(12), e2248812. 10.1001/jamanetworkopen.2022.48812. 10.1001/jamanetworkopen.2022.48812 PMC9856656 36580334

[28] National Center for Chronic Disease Prevention and Health Promotion. (2021, February). BRFSS statistical brief: Caregiver optional module. *Center for Disease Control and Prevention*. Retrieved August 23, 2023, from https://www.cdc.gov/aging/publications/BRFSS-caregiver-brief-508.pdf

[29] Otufowora, A., Liu, Y., Young, H., Egan, K. L., Varma, D. S., Striley, C. W., & Cottler, L. B. (2021). Sex differences in willingness to participate in research based on study risk level among a community sample of African Americans in North Central Florida. *Journal of Immigrant and Minority Health*, *23*(1), 19–25. 10.1007/s10903-020-01015-4 32328873 10.1007/s10903-020-01015-4 PMC7714285

[30] Milani, S. A., Cottler, L. B., & Striley, C. W. (2023). Perceptions of Research participation among a sample of Florida residents aged 50 and over reporting dementia. *Ageing International*, *48*(1), 95–107. 10.1007/s12126-021-09441-x 34483405 10.1007/s12126-021-09441-x PMC8406007

[31] Center for Behavioral Health Statistics and Quality (2018). *2019 National Survey on Drug Use and Health (NSDUH): CAI Specifications for Programming (English Version)*. Substance Abuse and Mental Health Services Administration. Retrieved from https://www.samhsa.gov/data/sites/default/files/reports/rpt23057/NSDUHmrbCAISpecs2019.pdf

[32] Scharff, D. P., Mathews, K. J., Jackson, P., Hoffsuemmer, J., Martin, E., & Edwards, D. (2010). More than Tuskegee: Understanding mistrust about research participation. *Journal of Health care for the poor and Underserved*, *21*(3), 879–897. 10.1353/hpu.0.0323 20693733 10.1353/hpu.0.0323 PMC4354806

[33] Hughes, T. B., Varma, V. R., Pettigrew, C., & Albert, M. S. (2017). African americans and Clinical Research: Evidence concerning barriers and facilitators to participation and recruitment recommendations. *The Gerontologist*, *57*(2), 348–358. 10.1093/geront/gnv118 26553736 10.1093/geront/gnv118 PMC6075213

[34] George, S., Duran, N., & Norris, K. (2014). A systematic review of barriers and facilitators to minority research participation among African Americans, latinos, Asian Americans, and Pacific Islanders. *American Journal of Public Health*, *104*(2), e16–e31. 10.2105/AJPH.2013.301706 24328648 10.2105/AJPH.2013.301706 PMC3935672

[35] Lang, R., Kelkar, V. A., Byrd, J. R., Edwards, C. L., Pericak-Vance, M., & Byrd, G. S. (2013). African American participation in health-related research studies: Indicators for effective recruitment. *Journal of Public Health Management and Practice*, *19*(2), 110–118. 10.1097/PHH.0b013e31825717ef 23358288 10.1097/PHH.0b013e31825717ef PMC10288523

[36] Hoyo, C., Reid, M. L., Godley, P. A., Parrish, T., Smith, L., & Gammon, M. (2003). Barriers and strategies for sustained participation of African-American men in cohort studies. *Ethnicity & Disease*, *13*(4), 470–476. 14632266

[37] Byrd, G. S., Edwards, C. L., Kelkar, V. A., Phillips, R. G., Byrd, J. R., Pim-Pong, D. S., … Pericak-Vance, M. (2011). Recruiting intergenerational African American males for biomedical research Studies: A major research challenge. *Journal of the National Medical Association*, *103*(6), 480–487. 10.1016/s0027-9684(15)30361-8. 10.1016/s0027-9684(15)30361-8 21830630

