# Supplementary material for: Exploring Trust in Research Among Black American Men at a Health Promotion Symposium in Rural North Carolina
Source: J Community Health. 2024 Sep 6;50(1):120–9. doi: 10.1007/s10900-024-01399-6 (PMC11805886; doi:10.1007/s10900-024-01399-6)
Supplement: Supplementary file 1 — Supplementary Material 1 [file 10900_2024_1399_MOESM1_ESM.docx]

**Figure 1 Data**

Have you ever participated in a health research study or clinical trial?

|  | Frequency | Percent |
| --- | --- | --- |
| No | 73 | 68.87 |
| Yes | 20 | 18.87 |
| No Response | 13 | 12.26 |

Are you interested in participating in a health research study or clinical trial?

|  | Frequency | Percent |
| --- | --- | --- |
| No | 57 | 53.77 |
| Yes | 32 | 30.19 |
| No Response | 17 | 16.04 |

**Figure 2 Data**

Willingness to Participate in Research Activities by Interest in Health Research Participation, n=106

|  | Interested in Research Participation  n=32 | | Not Interested in Research Participation  n=57 | | No Response  N=17 | |
| --- | --- | --- | --- | --- | --- | --- |
|  | Frequency | Percent | Frequency | Percent | Frequency | Percent |
| Complete a survey about your health* | 30 | 93.75 | 31 | 54.39 | 9 | 52.94 |
| Complete a memory and thinking test* | 29 | 90.63 | 35 | 61.40 | 10 | 58.82 |
| Have your medical records reviewed* | 28 | 87.50 | 30 | 52.63 | 10 | 58.82 |
| Give a blood sample* | 28 | 87.50 | 33 | 57.89 | 11 | 64.71 |
| Provide spinal fluid by having a needle inserted into your spinal canal (spinal tap) | 13 | 40.63 | 14 | 24.56 | 2 | 11.76 |
| Take medication* | 20 | 62.50 | 25 | 43.86 | 8 | 47.06 |
| Use medical equipment | 24 | 75.00 | 30 | 52.63 | 9 | 52.94 |
| Complete a brain scan* | 23 | 71.88 | 24 | 42.11 | 8 | 47.06 |
| Stay overnight in a hospital or clinic* | 23 | 71.88 | 23 | 40.35 | 10 | 58.82 |
| Be in a genetic study* | 23 | 71.88 | 23 | 40.35 | 6 | 35.29 |
| Donate your brain to research after death | 11 | 34.38 | 21 | 36.84 | 4 | 23.53 |
